# Aquatic Pilates Is Associated with Improved Spinal Alignment and Functional Performance in Women Aged 55–65 Years with Thoracic Kyphosis

**DOI:** 10.3390/jfmk11030371

**Published:** 2026-09-16

**Authors:** Paraskevi Passa, Vassilis Paschalis, Asimenia Gioftsidou, Helen Soultanakis

**Affiliations:** 1 School of Physical Education and Sport Science, National and Kapodistrian University of Athens, 17237 Athens, Greece; ppassa@phed.uoa.gr; 2 School of Physical Education and Sport Science, Democritus University of Thrace, 69100 Komotini, Greece; agioftsi@phyed.duth.gr; 3 Section of Human and Evolutionary Biology, Department of Biological Sciences, University of Southern California, Los Angeles, CA 90089, USA; elenisoul@hotmail.com

**Keywords:** thoracic kyphosis, aquatic Pilates, functional performance, women aged 55–65 years

## Abstract

**Objectives:** Thoracic kyphosis is a common postural condition associated with impaired functional performance, reduced balance, and lower levels of physical activity. Women may be particularly susceptible due to age-related musculoskeletal and hormonal changes. This study investigated the effects of a 12-week aquatic Pilates program on thoracic alignment, functional performance, and self-reported physical activity in women aged 55–65 years with thoracic kyphosis. **Methods:** Twenty women aged 55–65 years with thoracic kyphosis were randomly allocated to an Aquatic Pilates intervention group (*n* = 10) or a non-exercising control group (*n* = 10). Participants in the intervention group completed supervised aquatic Pilates sessions twice weekly for 12 weeks, while the control group maintained their usual daily activities. Pre- and post-intervention assessments included thoracic kyphosis angle measured using a DeBrunner kyphometer, the Modified Physical Performance Test (mPPT), and the Physical Activity Scale for the Elderly (PASE). Data were analyzed using 2 × 2 mixed-design repeated-measures ANOVA. The trial was registered retrospectively at ClinicalTrials.gov (NCT07674251). **Results:** A significant group × time interaction was observed for thoracic kyphosis angle, F_1,18_ = 66.31, *p* < 0.001, partial *η*^2^ = 0.786), with the Aquatic Pilates group showing a reduction from 42.6° ± 3.4° to 40.7° ± 3.1°. Significant improvements were also observed in the analyzed mPPT functional tasks (all *p* < 0.001). PASE scores showed a statistically significant group × time interaction (F_1,18_ = 9.48, *p* = 0.006, partial *η*^2^ = 0.345), with the Aquatic Pilates group showing an increase compared to the control group. **Conclusions:** Participation in a 12-week aquatic Pilates program was associated with improvements in thoracic kyphosis, functional performance as assessed by the mPPT, and self-reported physical activity in women aged 55–65 years with thoracic kyphosis.

## 1. Introduction

Thoracic kyphosis is the posterior convex curvature of the thoracic spine that contributes to upright posture and efficient load distribution during daily activities. With advancing age, this curvature often increases beyond normal limits, and kyphotic angles exceeding 40° have been reported in approximately 20–40% of adults older than 60 years [1,2]. Age-related reductions in muscle mass and strength, particularly in the trunk extensor musculature, along with decreases in bone mineral density, intervertebral disc degeneration, and reduced spinal mobility, may contribute to the progression of excessive thoracic kyphosis [3,4]. Women appear to be disproportionately affected, often developing kyphotic changes earlier and to a greater extent than men, potentially due to hormonal changes, lower bone density, connective tissue alterations, and reduced trunk extensor strength. Excessive thoracic kyphosis has been associated with impaired balance, reduced mobility, increased risk of falls, and lower levels of physical activity, highlighting the need for effective and targeted therapeutic strategies [5,6].

Exercise-based interventions are widely recommended to improve thoracic alignment and functional performance. Land-based exercise programs that include thoracic mobility, core stabilization, flexibility training, and postural retraining have demonstrated beneficial effects on spinal alignment, trunk control, and functional mobility in older adults [7,8,9]. Activation of deep stabilizing musculature, including the multifidus and transverse abdominis, appears to play an important role in postural correction and functional outcomes [10]. In addition, interventions that combine strengthening with proprioceptive and postural components may produce greater benefits than strengthening alone [11,12]. Recent systematic reviews have suggested that structured Pilates interventions may improve postural control, static and dynamic balance, and factors associated with fall risk in older adults, supporting the potential value of movement programs that emphasize neuromuscular coordination [13,14].

Aquatic exercise represents a promising modality for rehabilitation and functional enhancement in aging populations because of the unique mechanical properties of water, including buoyancy, viscosity, and hydrostatic pressure. These properties reduce joint loading while facilitating movement, improving mobility, and promoting neuromuscular activation [15]. Previous evidence suggests that water-based training may improve flexibility, muscular endurance, balance, and functional mobility in older adults, including those with postural impairments or mobility limitations [16,17]. However, limited research has examined aquatic Pilates specifically in relation to thoracic alignment and functional performance in women with thoracic kyphosis.

Pilates-based exercise emphasizes core stabilization, controlled breathing, spinal alignment, and postural awareness, and has been associated with improvements in trunk strength, movement control, and balance in older adults [13,18]. Aquatic Pilates combines Pilates-based exercises with the mechanical properties of the aquatic environment. However, the extent to which this specific exercise approach is associated with changes in thoracic alignment and functional performance remain unclear.

Therefore, the aim of the present study was to investigate whether participation in a 12-week aquatic Pilates program was associated with changes in thoracic alignment, functional performance, and self-reported physical activity (assessed via PASE) in women aged 55–65 years with thoracic kyphosis. It was hypothesized that participants in the Aquatic Pilates group would demonstrate greater improvements in these outcomes than participants in the no-exercise control group. Physical activity was assessed to examine whether participation in the intervention was accompanied by changes in overall self-reported physical activity, rather than assuming an automatic increase in habitual physical activity outside the supervised sessions.

## 2. Materials and Methods

### 2.1. Participants

A total of 20 women aged 55–65 years were assessed for eligibility, and all met the inclusion criteria. Consequently, all 20 participants were enrolled, randomly allocated in a 1:1 ratio to either the Aquatic Pilates group (*n* = 10) or the no-exercise control group (*n* = 10), and successfully completed the 12-week intervention and post-intervention assessments (100% completion rate, 0% dropout). Thus, no participants were lost to follow-up, and all 20 randomized participants were included in the final statistical analysis.

This participant flow is embedded within the broader pre-planned four-arm study protocol, where a total of 40 women aged 55–65 years were screened across all four study arms (Aquatic Pilates, Land-based Pilates, Women without kyphosis, and Non-exercise controls; *n* = 10 per group). All 40 screened individuals met the inclusion criteria, agreed to participate, and were randomized (100% eligibility and enrollment rate; 0 individuals excluded prior to or post-randomization, 0% dropouts across the total sample). Eligibility criteria included women aged 55–65 years with a thoracic kyphosis angle between 40° and 46°. The baseline threshold of 40° was selected as it represents the clinically accepted cutoff for age-related hyperkyphosis associated with impaired balance and musculoskeletal functional decline [1,2]. The upper limit of 46° was applied to ensure a homogeneous sample of mild-to-moderate, reducible postural kyphosis suitable for group-based aquatic exercise. To distinguish reducible postural kyphosis from rigid structural deformities (such as Scheuermann’s disease, severe osteoporosis, or prior vertebral compression fractures), all participants underwent baseline clinical postural and spinal mobility screening. Screening included physical assessment of thoracic extension and curve reducibility, alongside a medical history review to confirm the absence of spinal fractures, spinal surgery within the preceding 6 months, rheumatoid arthritis, chronic obstructive pulmonary disease, or cardiovascular disease.

The overall sample had a mean ± SD: age 58.8 ± 1.2 yrs; body weight 61.5 ± 3.0 kg; height 165 ± 3.0 cm; BMI 22.6 ± 1.8 kg/m^2^. Participants in the Aquatic Pilates group had a baseline thoracic kyphosis angle of 42.6° ± 3.4° and participated in the 12-week aquatic Pilates program. Participants in the no-exercise control group had a baseline thoracic kyphosis angle of 43.0° ± 4.9° and were instructed to maintain their usual daily activities and refrain from participating in any structured exercise program throughout the study period (Table 1).

The random allocation sequence was generated using a computerized randomization procedure with a 1:1 ratio. Allocation concealment was implemented using sequentially numbered, sealed envelopes opened immediately before group assignment. The researcher responsible for participant enrollment also performed the allocation. Because of the nature of the intervention, blinding of participants and intervention personnel was not feasible. Eligible participants attended an informational session describing the study objectives, assessment procedures, and intervention protocol. The study was approved by the Institutional Ethics Committee (Approval No. 1402/13-07-2022) and conducted in accordance with the Declaration of Helsinki for research involving human participants. The trial was registered retrospectively at ClinicalTrials.gov (NCT07674251). The study commenced on 2 January 2025 and was completed on 27 March 2025; the trial record was first submitted to ClinicalTrials.gov on 13 June 2026 and first posted on 29 June 2026. Written informed consent was obtained from all participants prior to enrollment.

### 2.2. Intervention

The intervention consisted of a 12-week aquatic Pilates program designed to improve thoracic alignment, core stability, and postural control. The program incorporated exercises targeting the abdominal, spinal, and pelvic musculature through a combination of stabilizing and dynamic movements involving multiple muscle groups. Breathing control was emphasized throughout all sessions to promote concentration, relaxation, movement efficiency, and postural awareness. Functional exercises were also incorporated to promote coordinated core-to-limb activation, with the goal of improving balance, flexibility, and muscular endurance. Participants were encouraged to perform all movements with controlled and efficient body mechanics to support a stable foundation during daily activities [19,20].

The program consisted of 24 supervised sessions delivered twice weekly over 12 weeks, with each session lasting 45 min. Exercise intensity was continuously monitored at the middle and end of each session using the Borg Rating of Perceived Exertion (RPE 6–20) scale, maintaining a light-to-moderate level between 11 (‘light’) and 13 (‘somewhat hard’), in accordance with ACSM guidelines for older adults [21]. All sessions were conducted in an indoor swimming pool under controlled environmental conditions. Water temperature was maintained between 30–32 °C throughout the intervention period to ensure participant comfort and facilitate muscle relaxation. Given the pool depth of 1.20–1.30 m, water immersion reached approximately the chest level (xiphoid process) for all participants, providing an estimated body weight unloading of at least 60% while ensuring stable footholds during exercise [22]. The aquatic environment was selected to utilize the biomechanical properties of water, including buoyancy and hydrostatic pressure, which reduce joint loading and facilitate the safe performance of postural and core-focused exercises (Table 2). All sessions were supervised and conducted by the researcher, a certified Pilates instructor experienced in aquatic exercise with a BA in aquatics and sports science. Compliance with the intervention was 100%, with all participants completing all 24 scheduled sessions (no missed sessions requiring make-up classes). Furthermore, no adverse events, injuries, muscle soreness requiring rest, or other side effects were reported throughout the 12-week study period.

Participants in the control group were instructed to maintain their usual daily activities and refrain from participating in any structured exercise program during the study period. Compliance with these instructions was monitored through participant self-report during the study period. Participants were asked to report any participation in structured exercise, Pilates, or rehabilitation programs. No participant in the control group reported initiating or participating in a new structured exercise, Pilates, or rehabilitation program during the 12-week intervention.

### 2.3. Outcome Measures

Anthropometric characteristics, including body weight, height, and body mass index (BMI), were collected for all participants at baseline. Thoracic kyphosis was assessed using a mechanical Debrunner’s kyphometer (Protek, AG, Bern, Switzerland), with participants standing in their relaxed upright posture. The T2 and T12 anatomical landmarks were identified using the same standardized landmark-identification procedure throughout the study, and the kyphometer was positioned to determine the thoracic kyphosis angle across the T2–T12 region. Three measurements were obtained at each assessment time point, and the mean of the three readings was used for statistical analysis. All measurements were performed by the same researcher using the same standardized procedure. The assessor was not blinded to group allocation because participants’ intervention status was known during the assessment period.

A preliminary within-session repeatability assessment was conducted using the same T2–T12 measurement procedure and assessor. This assessment yielded an ICC (3,1) of 0.983, an SEM of 0.46°, and an MDC95 of 1.27°. Because these estimates were obtained from a small preliminary sample, they should be interpreted cautiously.

Physical function was assessed using the 9-item Modified Physical Performance Test (mPPT) [23], with a total scoring range from 0 to 36 points (higher scores indicating better performance). The 9 items included lifting a weighted object, donning/removing a jacket, picking up a small object, walking 15.24 m, 5 chair rises, turning 360°, static standing balance (feet together, semi-tandem, full tandem), climbing one flight of stairs, and climbing four flights of stairs. Each subtest is scored on a scale from 0 to 4 based on completion time or performance criteria.

In addition to the total mPPT score, the completion time for each individual task was analyzed separately to provide a more detailed assessment of changes in specific functional activities. The assessed tasks included lifting a weighted object, donning and removing a jacket, picking up a small object from the floor, walking a 15.24 m course, performing five consecutive chair rises without using the arms, turning 360°, maintaining static standing balance in progressively challenging stances, and stair climbing. All assessments were performed according to standardized mPPT procedures and were timed by trained assessors.

Physical activity levels were assessed using the Physical Activity Scale for the Elderly (PASE) questionnaire, a validated self-report questionnaire used to quantify leisure, household, and occupational activity in older adults [24]. All outcome measurements were performed by the researcher at baseline and post-intervention using standardized assessment protocols.

### 2.4. Statistical Analysis

Data are presented as mean ± standard deviation (SD). Statistical analyses were performed using SPSS version 23 (IBM Corp., Armonk, NY, USA). A 2 × 2 mixed-design repeated-measures ANOVA was used to examine the effects of group (Aquatic Pilates vs. no-exercise control) and time (pre-intervention vs. post-intervention), as well as the group × time interaction.

Normality of data distribution was assessed using the Shapiro–Wilk test in combination with visual inspection of Q-Q plots. Mauchly’s test confirmed sphericity for repeated measures and Levene’s test confirmed homogeneity of variance across groups (*p* > 0.05). Box’s M test was used to confirm the equality of covariance matrices. For outcome hierarchy and multiplicity management, thoracic kyphosis angle was pre-specified as the single primary outcome. Functional performance (mPPT total score) and self-reported physical activity (PASE) were analyzed as secondary outcomes, whereas completion times for individual mPPT tasks were treated as exploratory outcomes. To control Type I error inflation due to multiple testing, Bonferroni adjustments were applied to post hoc pairwise comparisons, and secondary/exploratory outcomes were interpreted strictly within a hypothesis generating context without formal multi-endpoint adjustment. Baseline demo-graphic, anthropometric, postural, and functional parameters were evaluated descriptively; Baseline parameters are presented descriptively in Table 1 and Table 3. Effect sizes are reported as partial eta squared *η*^2^_p_ and interpreted as small (0.01), medium (0.06), or large (0.14). Statistical significance was set at *p* < 0.05.

An a priori sample-size calculation was performed using G*Power version 3.1 for the broader four-group study protocol, based on the within–between interaction in a repeated-measures ANOVA. Assuming a large effect size (f = 0.40), an alpha level of 0.05, and 80% statistical power, the calculation indicated a minimum total sample size of 36 participants. The recruitment target was therefore set at 40 participants, allowing for 10 participants per group in the broader study design.

The present manuscript reports a focused two-group analysis derived from a broader, pre-planned four-group randomized controlled study design (which originally included: (1) Aquatic Pilates, (2) Land-based Pilates, (3) Women without kyphosis and (4) No-exercise Control; total planned *n* = 40, 10 per group). The primary objective of this specific publication was to isolate and evaluate the distinct efficacy of the Aquatic Pilates intervention against an inactive control baseline. Importantly, the primary outcome (thoracic kyphosis angle) and secondary outcomes (mPPT scores and PASE physical activity) for this pairwise comparison were pre-specified in the original research protocol prior to data collection.

## 3. Results

Repeated measures ANOVA revealed significant time × group interactions across several outcome measures, indicating that changes over time differed between the Aquatic Pilates and non-exercising control groups.

A significant time × group interaction was observed for thoracic kyphosis (F_1,18_ = 66.31, *p* < 0.001, partial *η*^2^ = 0.786). Participants in the Aquatic Pilates group demonstrated a reduction in thoracic kyphosis following the 12-week intervention (42.6° ± 3.4° to 40.7° ± 3.1°), whereas the control group remained unchanged (43.0° ± 4.9° to 43.0° ± 4.9°). The between-group difference in mean change was −1.90° (95% CI: −2.25° to −1.55°) (Table 1).

Analysis of the Modified Physical Performance Test total score revealed a significant group × time interaction ((F_1,18_ = 227.12, *p* < 0.001, partial *η*^2^ = 0.927), indicating that overall functional performance improved in the Aquatic Pilates group over the 12-week intervention (24.3 ± 0.5 to 28.7 ± 0.8), whereas the control group remained relatively stable (24.0 ± 0.0 to 23.8 ± 0.4). The between-group difference in mean change was +4.60 (95% CI: 4.02 to 5.18 points) (Table 1).

Regarding self-reported physical activity, a significant group × time interaction was observed for PASE scores, F_1,18_ = 9.48, *p* = 0.006, partial *η*^2^ = 0.345. PASE scores increased in the Aquatic Pilates group and decreased in the no-exercise control group. Between-group difference in change was 24.93 PASE points (95% CI, 7.91 to 41.94). However, this finding should be interpreted cautiously because the PASE assesses self-reported activity during the preceding seven days and may partly reflect participation in the supervised intervention rather than a sustained increase in habitual physical activity outside the intervention (Table 1).

**Figure 1:**
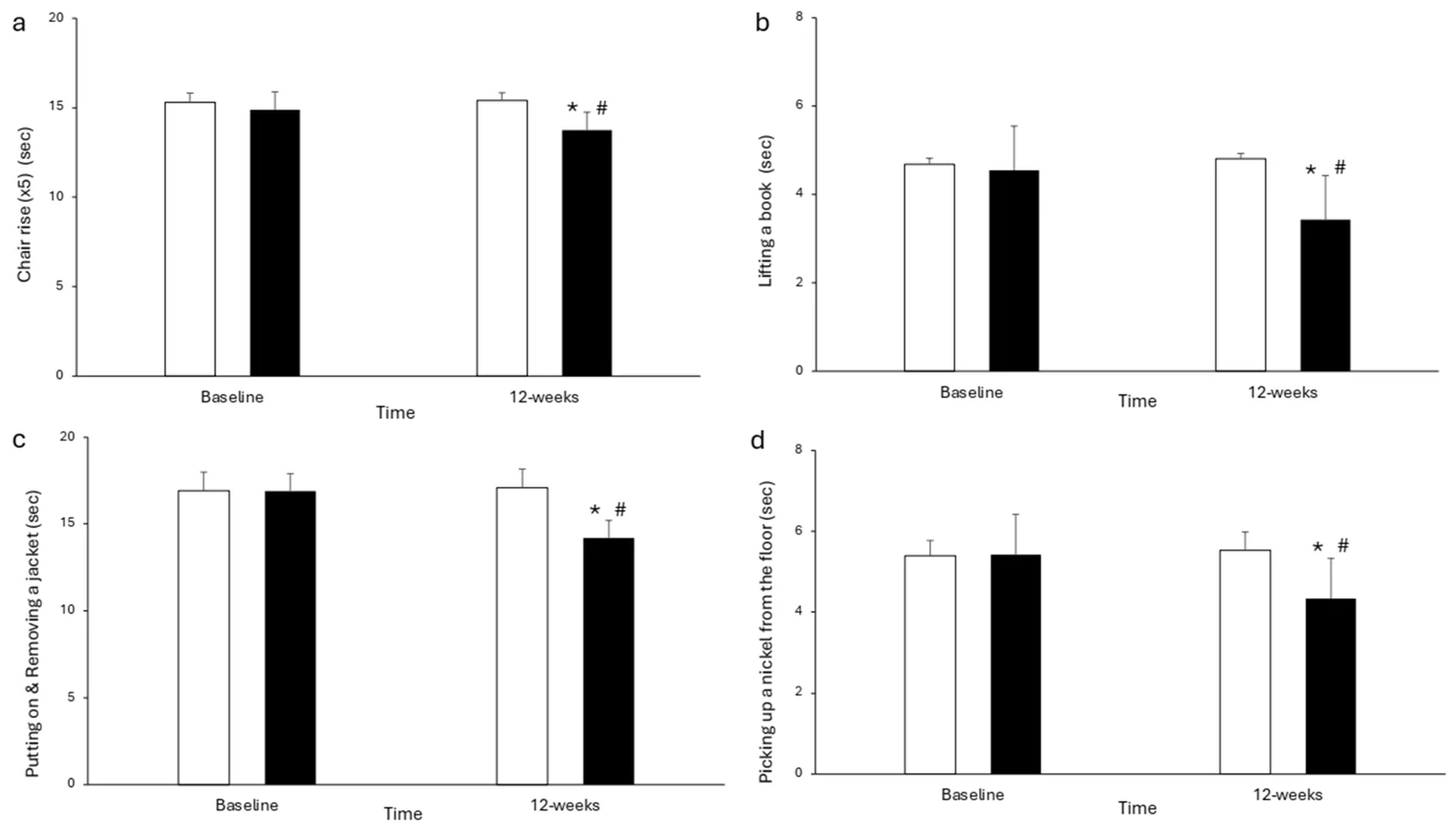
Changes in functional task performance from baseline to 12 weeks in Aquatic Pilates (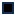) and control (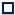) groups: (**a**) Chair rise test (5 repetitions) without arm support; (**b**) Lifting a book and placing it on a shelf; (**c**) Putting on and removing a jacket; (**d**) Picking up a coin from the floor. * Significant difference from baseline for the same group (*p* < 0.05). # Significant difference between the two groups at the same time point (*p* < 0.05).

**Figure 2:**
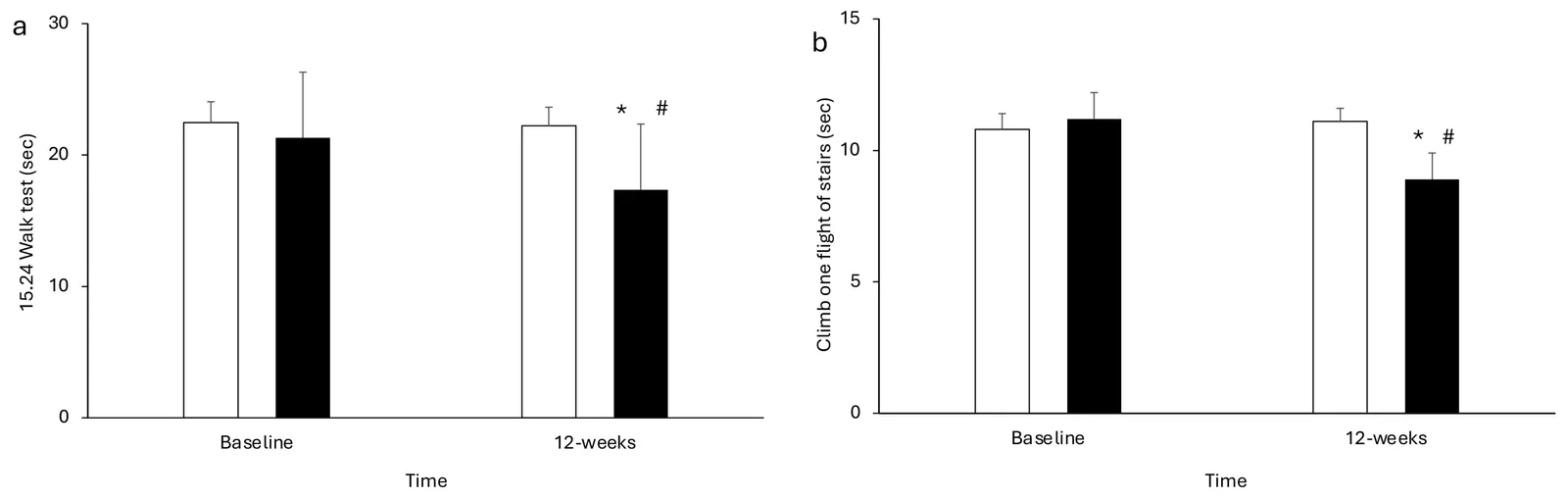
Changes in mobility performance from baseline to 12 weeks in Aquatic Pilates (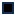) and control (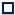) groups: (**a**) 15.24-m walk test; (**b**) Stair climb test (one flight of stairs). * Significant difference from baseline for the same group (*p* < 0.05). # Significant difference between the two groups at the same time point (*p* < 0.05).

Significant time × group interactions were also observed for functional performance tasks. Participants in the Aquatic Pilates group demonstrated significant reductions in completion times for lifting a weighted object, donning and removing a jacket, picking up a small object, walking a 15.24 m course, performing five consecutive chair rises, and climbing one flight of stairs (all *p* < 0.001). In contrast, performance in the control group remained stable in all tasks (Figure 1 and Figure 2). For three of the mPPT subtests (turning 360°, static standing balance, and climbing four flights of stairs), all participants in both groups achieved the maximum possible score (4.0 ± 0.0) at both baseline and 12 weeks, demonstrating a ceiling effect. Consequently, overall mPPT total score improvements were driven by the remaining six timed functional tasks. Detailed functional performance outcomes, including between-group difference in changes and 95% Confidence Intervals, are presented in Table 3.

Statistical assumptions were confirmed: normality via Shapiro–Wilk test (*p* > 0.05), sphericity via Mauchly’s test, and homogeneity of variances/covariances via Levene’s (*p* > 0.05) and Box’s M tests. 

## 4. Discussion

The present study examined the effects of a 12-week aquatic Pilates program on thoracic alignment, functional performance, and self-reported physical activity in women aged 55–65 years with thoracic kyphosis. The main findings were that participants in the Aquatic Pilates group demonstrated a reduction in thoracic kyphosis angle (~1.9°), and improvements across multiple functional tasks, whereas the no-exercising control group remained largely unchanged. PASE scores increased in the Aquatic Pilates group and decreased in the control group, resulting in a statistically significant group × time interaction. However, this finding should be interpreted cautiously, as the PASE questionnaire assesses physical activity during the preceding seven days and may partly reflect participation in the supervised intervention sessions rather than an increase in habitual physical activity in daily life.

The observed mean reduction in thoracic kyphosis of approximately 1.9° was statistically significant and exceeded MDC95 estimate of 1.27° derived from our within-session repeatability assessment. However, because this repeatability estimate was obtained from a small preliminary sample and no established minimal clinically important difference (MCID) for the specific DeBrunner kyphometer T2–T12 protocol, the observed change should be characterized as a statistically significant reduction in postural alignment rather than an established clinically meaningful improvement. Nevertheless, excessive thoracic kyphosis has been associated with altered spinal biomechanics, anterior displacement of the center of mass, increased muscular demands during upright posture, impaired balance, reduced mobility, and an increased risk of falls [1,3,5]. The reduction in thoracic kyphosis angle is consistent with previous evidence indicating that targeted corrective and spinal-extension exercises can improve thoracic posture. Seidi et al. [12] reported beneficial effects of corrective exercise interventions on thoracic hyperkyphosis, while Sinaki et al. [3] demonstrated that a spinal proprioceptive extension exercise program reduced fall and back-pain risk among women with osteoporotic kyphosis. The present study extends these findings by suggesting that Pilates-based exercise performed in an aquatic environment may also improve thoracic alignment. The emphasis of Pilates on axial elongation, trunk stabilization, postural awareness, and controlled movement may have contributed to the observed reduction in thoracic kyphosis angle [8,19,20]. Direct comparisons with previous studies should nevertheless be made cautiously because of differences in participant characteristics, exercise modalities, intervention duration, and methods used to assess thoracic curvature.

Functional performance improvements were observed across all mPPT tasks, including lifting, dressing, reaching, walking, chair rises, and stair climbing. These activities represent critical daily functions and suggest that aquatic Pilates provides a multi-dimensional enhancement of strength, mobility, and neuromuscular coordination [4,10]. These findings are consistent with systematic reviews indicating that Pilates interventions can improve balance and physical performance and reduce factors associated with fall risk in older adults [6,13,14,18]. They also align with evidence that aquatic exercise can improve physical performance, balance, gait, and muscular strength in older populations [2,4,9,10]. These improvements may reflect the integrated effects of Pilates principles and the physical properties of the aquatic environment. However, the present study design does not permit the relative contributions of the individual intervention components to be determined.

The present findings therefore contribute to existing literature by demonstrating concurrent improvements across several functional tasks following an aquatic Pilates intervention. The concurrent improvement in spinal alignment and functional performance may reflect the combined influence integrated effects of Pilates principles and the physical properties of the aquatic environment; However, the present study design does not allow the independent effects of the different exercise components of the intervention to be determined. Potential mechanisms may include increased activation of trunk stabilizers, enhanced proprioceptive input through water resistance, and reduced axial loading due to buoyancy, facilitating safe performance of corrective postural movements [4,6,7,8,10]. Additionally, the supportive aquatic environment may enhance participants’ movement confidence and exercise adherence. This is consistent with the statistically significant group × time interaction observed for self-reported physical activity (F_1,18_= 9.48, *p*= 0.006, partial *η*^2^ = 0.345), where PASE scores increased in the Aquatic Pilates group compared to the control group. However, as noted, this finding should be interpreted cautiously, as the PASE assesses activity during the preceding seven days and may partially reflect attendance in the supervised intervention sessions rather than a permanent increase in habitual physical activity outside the program. However, these mechanisms were not directly assessed in the present study and therefore remain speculative. 

### Limitations

Several limitations should be considered when interpreting these findings. The study included 20 women aged 55–65 years with thoracic kyphosis; therefore, the extent to which the findings apply to broader populations remains to be established. In addition, although participants were randomly allocated to the Aquatic Pilates or non-exercising control group, the original sample-size calculation was based on the broader study design rather than specifically on the two-group analysis presented here. The assessor was aware of group allocation, and thoracic kyphosis was evaluated using a clinical kyphometer rather than radiographic imaging. Furthermore, the absence of an active exercise comparator prevents the effects of Pilates from being distinguished from those of the aquatic environment or their combined influence, while the lack of long-term follow-up precludes conclusions regarding the maintenance of the observed improvements. Participant-reported measures may have been susceptible to reporting bias. Specifically, regarding functional performance, a ceiling effect was present for three mPPT subtests (turning 360°, static standing balance, and climbing four flights of stairs), as all participants in both groups achieved maximum scores (4.0 ± 0.0) at baseline and 12 weeks, thereby limiting the sensitivity to detect potential improvements in these specific domains. Although adjusted pairwise comparisons were applied, the evaluation of multiple outcomes may have increased the risk of type I error. Finally, the kyphometer repeatability assessment was based on three individuals, and the physiological mechanisms underlying the observed changes were not directly examined. Future randomized controlled trials incorporating larger and more diverse samples, active comparator groups, blinded outcome assessment where feasible, and longer-term follow-up would help extend and confirm these findings.

## 5. Conclusions

A structured 12-week aquatic Pilates program was associated with reductions in thoracic kyphosis angle and improvements in functional performance and self-reported physical activity in women aged 55–65 years with thoracic kyphosis. These findings indicate that aquatic Pilates represents a feasible exercise modality associated with improvements in postural alignment and functional performance in women aged 55–65 years with thoracic kyphosis. However, because the intervention was compared against an inactive control group, the specific therapeutic effect of the aquatic environment relative to land-based exercise cannot be confirmed. Future research involving larger cohorts, long-term follow-up assessments, and active comparison groups (such as land-based Pilates) is warranted.

## Figures and Tables

**Table 1:** Anthropometric Characteristics, Kyphosis angle, Modified Physical Performance Test (mPPT) total score and PASE at baseline and after 12 weeks in No-exercise Group and Aquatic Pilates Group. * Significantly different from baseline within the Aquatic Pilates group (*p* < 0.05). F values represent the group × time interaction. No significant changes were detected in the No-exercise Group.

	Aquatic Pilates Group Baseline	Aquatic Pilates Group 12 Weeks	No-Exercise Group Baseline	No-Exercise Group 12 Weeks	Between-Group Change Difference (95% CI)	F (1,18)	*p*	Partial *η*^2^
Age (y)	58.6 ± 1.2	-	58.9 ± 1.1	-	-	-	-	-
Height (cm)	165.7 ± 2.6	-	163.5 ± 3.4	-	-	-	-	-
Weight (kg)	61.6 ± 3.3	60.6 ± 2.6	61.3 ± 2.8	61.6 ± 2.6	-	-	-	-
BMI	22.1 ± 2.6	22.1 ± 1.1	23.0 ± 1.7	23.1 ± 1.6	-	-	-	-
Kyphosis angle (°)	42.6 ± 3.4	40.7 ± 3.1 *	43.0 ± 4.9	43.0 ± 4.9	−1.90 (−2.25, −1.55)	66.31	<0.001	0.786
mPPT	24.3 ± 0.5	28.7 ± 0.8 *	24.0 ± 0.0	23.80 ± 0.4	+4.60 (4.02, 5.18)	227.12	<0.001	0.927
PASE	98.2 ± 19.2	115.5 ± 17.1 *	94.5 ± 19.2	86.9 ± 17.2	+24.93 (7.91, 41.94)	9.48	0.006	0.345

Note: Hyphens (-) denote variables (age and height) that were not re-assessed at 12 weeks because they do not change over the study period. Zero variance in the control group baseline mPPT total score (24.0 ± 0.0) reflects identical scores across all participants due to ceiling effects on specific items.

**Table 2:** Indicative 12-week water-based Pilates exercise program used in the intervention (2 sessions/week, 45 min/session) for women aged 55–65 years with thoracic kyphosis.

Phase 1: Familiarization & Stabilization	Weeks: 1–4	Duration 45 min
Deep Breathing in Water	5 breaths	Improve breathing awareness and thoracic expansion
Shoulder Rolls & Scapula Squeeze	2 × 10	Shoulder mobility, posture improvement
Chest Opener with Float	2 × 5–8 s hold	Stretch thorax and shoulder
Water March (standing)	2 min	Improve balance and lower body mobility
Arm Circles with Water Resistance	2 × 10 forward/back	Shoulder strengthening, increase range of motion
Seated Torso Twist	2 × 8 each side	Controlled trunk rotation
Standing Leg Lift	2 × 8 each leg	Strengthen glutes and hip muscles
Phase 2: Strength & Mobility	Weeks: 5–8	Duration 45 min
Water March with Arm Resistance	2–3 min	Core activation, cardiovascular activity
Standing Back Extension with Float	2 × 10	Strengthen back muscles, improve kyphosis
Single Leg Kick on Water Platform	2 × 10 each leg	Lower body strength, pelvic stabilization
Arm Float Press	2 × 12	Strengthen shoulders and arms
Side Leg Lift with Float	2 × 8–10 each side	Strengthen glutes and lateral abdominals
Seated Spinal Rotation with Float	2 × 8 each side	Spine flexibility
Pelvic Curl in Water	2 × 10	Core stabilization, lower body mobility
Standing Posture Alignment Drill	1–2 min	Correct kyphosis, focus on posture
Phase 3: Stabilization & Balance	Weeks: 9–12	Duration 45 min
Water Walking with Arm Resistance	3 min	Core strengthening, cardiovascular activity
Standing Back Extension with Float & Light Resistance	2 × 12	Strengthen back muscles
Side Kick with Float	2 × 10 each side	Strengthen lateral abdominals and glutes
Arm Sweep & Circle Combo	2 × 10	Coordination, shoulder strengthening
Seated Torso Curl with Float	2 × 10	Strengthen abdominals, core stabilization
Pelvic Tilt + Bridge in Water	2 × 10	Core stabilization, pelvic mobility
Balance on One Leg with Float Support	30 s each leg	Improve balance, ankle/hip stabilization

Note. Each session followed the same structure across all phases: warm-up (5 min) including deep breathing with arm elevation in water, shoulder rolls (forward/backward), and head and pelvic rotations; main session (35 min) consisting of strengthening, flexibility, and core stabilization exercises performed in various positions (standing, seated, side-lying, or using floats/straps); and cool-down (5 min) including stretching exercises for the back, shoulders, and neck as well as gentle breathing techniques.

**Table 3:** Functional performance test results at baseline and after the 12-week intervention (mean ± SD). * Significant different from baseline within the Aquatic Pilates group (*p* < 0.05). F values represent the group × time interaction.

Test	Aquatic Pilates Group Baseline	Aquatic Pilates Group 12 Weeks	No-Exercise Group Baseline	No-Exercise Group 12 Weeks	Between-Group Change Difference (95% CI)	F (1,18)	*p*	Partial *η*^2^
Lifting a weighted object (s)	4.55 ± 0.14	3.43 ± 0.12 *	4.68 ± 0.14	4.81 ± 0.12	−1.25 (−1.59, −0.91)	59.17	<0.001	0.77
Donning/removing a jacket (s)	16.53 ± 1.30	14.20 ± 1.13 *	16.91 ± 1.08	17.09 ± 1.08	−2.50 (−2.86, −2.15)	211.66	<0.001	0.92
Picking up small object (s)	5.42 ± 0.28	4.33 ± 0.30 *	5.39 ± 0.38	5.53 ± 0.45	−1.23 (−1.47, −0.99)	117.87	<0.001	0.87
Walking 15.24 m (s)	21.31 ± 1.04	17.36 ± 0.79 *	22.48 ± 1.58	22.22 ± 1.42	−3.69 (−4.25, −3.14)	196.01	<0.001	0.92
Chair rises ×5 (s)	14.89 ± 0.51	13.75 ± 0.29 *	15.27 ± 0.52	15.36 ± 0.44	−1.23 (−1.51, −0.95)	88.19	<0.001	0.83
One-flight stairs (s)	11.22 ± 0.64	8.89 ± 1.03 *	10.82 ± 0.50	11.02 ± 0.52	−2.53 (−2.88, −2.18)	237.34	<0.001	0.93

Three mPPT subtests (turning 360°, static standing balance, and climbing four flights of stairs) are omitted from the table because all participants in both groups achieved the maximum possible score (4.0 ± 0.0) at both baseline and 12 weeks (ceiling effect), resulting in zero variance and no statistically detectable change.

## Data Availability

The data supporting the findings of the study are available from the corresponding author upon reasonable request. The data are not publicly available due to ethical restrictions and the need to protect participant confidentiality.

## Abbreviations

The following abbreviations are used in this manuscript:

ACSM	American College of Sports Medicine
BMI	Body Mass Index
mPPT	Modified Physical Performance Test
PASE	Physical Activity Scale for the Elderly
SD	Standard Deviation
SPSS	Statistical Package for the Social Sciences
